# Soybean leaf estimation based on RGB images and machine learning methods

**DOI:** 10.1186/s13007-023-01023-z

**Published:** 2023-06-17

**Authors:** Xiuni Li, Xiangyao Xu, Shuai Xiang, Menggen Chen, Shuyuan He, Wenyan Wang, Mei Xu, Chunyan Liu, Liang Yu, Weiguo Liu, Wenyu Yang

**Affiliations:** 1 College of Agronomy, Sichuan Agricultural University, Chengdu, China; 2 Sichuan Engineering Research Center for Crop Strip Intercropping System, Chengdu, China; 3 Key Laboratory of Crop Ecophysiology and Farming System in Southwest, Ministry of Agriculture, Chengdu, China

**Keywords:** Soybean, Leaf parameters, Estimation, RGB, Machine learning

## Abstract

**Background:**

RGB photographs are a powerful tool for dynamically estimating crop growth. Leaves are related to crop photosynthesis, transpiration, and nutrient uptake. Traditional blade parameter measurements were labor-intensive and time-consuming. Therefore, based on the phenotypic features extracted from RGB images, it is essential to choose the best model for soybean leaf parameter estimation. This research was carried out to speed up the breeding procedure and provide a novel technique for precisely estimating soybean leaf parameters.

**Results:**

The findings demonstrate that using an Unet neural network, the IOU, PA, and Recall values for soybean image segmentation can achieve 0.98, 0.99, and 0.98, respectively. Overall, the average testing prediction accuracy (ATPA) of the three regression models is Random forest > Cat Boost > Simple nonlinear regression. The Random forest ATPAs for leaf number (LN), leaf fresh weight (LFW), and leaf area index (LAI) reached 73.45%, 74.96%, and 85.09%, respectively, which were 6.93%, 3.98%, and 8.01%, respectively, higher than those of the optimal Cat Boost model and 18.78%, 19.08%, and 10.88%, respectively, higher than those of the optimal SNR model.

**Conclusion:**

The results show that the Unet neural network can separate soybeans accurately from an RGB image. The Random forest model has a strong ability for generalization and high accuracy for the estimation of leaf parameters. Combining cutting-edge machine learning methods with digital images improves the estimation of soybean leaf characteristics**.**

**Supplementary Information:**

The online version contains supplementary material available at 10.1186/s13007-023-01023-z.

## Background

Today, soybeans are an important crop for grain, oil, and feed. The soybean planting area is second only to cash food crops such as wheat, rice, and maize, while the trade volume ranks first among various agricultural products [1]. It is especially crucial to develop high-yield soybean varieties because, according to statistics from the US Department of Agriculture, China imports up to 85% of its soybeans each year, and its average yield is only 132.4 kg/mu, much lower than the global average of 188.7 kg/mu. To assess vegetation growth dynamics and crop productivity, leaves have been widely investigated for many years. They have a direct impact on sunlight penetration, light absorption, CO_2_ fixation, and photosynthetic efficiency [2].

In recent years, phenotypic research has primarily focused on leaf number as a key phenotypic attribute, which is a vital morphological metric used to assess crop development and canopy structure [3]. For example, in maize, the number of leaves is correlated with plant height, flowering time, and moisture at harvest [4]. A greater number of leaves in switchgrass, indicative of a longer duration of vegetative growth prior to reproductive transition, is highly correlated to biomass yield [5]. In tobacco, the number of leaves closely relates to the quality of the tobacco leaves, and a reasonable number of leaves ensures high-quality tobacco leaves [6]. In potato, the number of green leaves has been used as an indicator to determine drought-resistant and susceptible varieties [7]. The number of leaves in perennial ryegrass is used as a criterion for determining defoliation time [8]. The number of leaves on soybean plants is a crucial indicator for determining the vegetative growth stage's growth period, which can be used to adjust the sowing date, choose the peak control period, and determine when to apply herbicides. Therefore, plant biologists, plant breeders, and agronomists often count the number of leaves on a particular plant.

Leaf fresh weight (LFW) is a critical indicator for assessing crop growth since it is directly related to biomass and dry matter buildup. One of the key markers for assessing the drought and cold tolerance of maize is its LFW [9, 10]. In tobacco, when approaching maturity, the LFW should be strictly controlled to prevent the tobacco leaves from turning green. In wheat, cotton, soybean and other crops, the fresh weight of leaves should be controlled in time after entering the reproductive growth stage to prevent excessive leaf growth and nutrient waste.

The leaf area index (LAI), defined as the photosynthetically active area per unit horizontal surface area, is related to crop development, water use, nutrient uptake, and yield [11–13] and used to monitor changes in canopy structure and assess environmental adaptability [14, 15]. The LAI is also used in crop breeding and production to monitor crop growth and estimate yield [16]. As a result, leaf characteristics are crucial indicators in soybean breeding. According to a literature review, there are two frequently used approaches (direct and indirect) for monitoring soybean leaves. In soybean breeding, leaf characteristics are typically disregarded since direct methods are more accurate than indirect methods, but they also take longer and are frequently destructive. Thus, how can soybean leaves be monitored accurately and efficiently to support effective soybean breeding?

Precision agriculture has become a popular topic in recent years, and the development of nondestructive estimation technologies have provided new methods and means for crop growth estimation, presenting good application prospects. Previous studies have shown that images collected by sensors such as an RGB camera [17], a thermal infrared camera [18], a hyperspectral camera [19], and a CT scanner [20] can extract multiple image traits, and based on these image traits, prediction models of the leaf area index [21], leaf iron deficiency greening, and other indicators [22, 23] can be established. Among the above-mentioned sensors, thermal infrared cameras work in the field environment, which is greatly affected by the ambient temperature and has an extremely low resolution [24]. Although hyperspectral cameras have many continuous bands and can acquire spectral images with numerous bands, image processing takes a long time because of the quantity of the information contained. CT scanners are expensive and challenging to use. The advantages of RGB cameras over other image acquisition tools include their ease of use, low cost, broad application range, and simple operation. Therefore, in the past 10 years, researchers have worked hard to develop RGB camera applications for soybean leaves.

Reports identify two widely used techniques for automatically counting blades: regression counting based on comprehensive picture analysis and detection counting. Miao C. et al. [25] show that both methods achieve RMSE (root mean square error) less than a single leaf and only slightly lower than the human-annotated RMSE (between 0.57 and 0.73 leaves). These methods have been studied for crops like maize [25], sorghum [25], and Arabidopsis thaliana [26]. However, the regression calculation approach based on convolutional neural networks (CNNs) underestimates the extreme leaf number of plants in the dataset, with low accuracy and increasing bias.

Scientists prefer to separate and count the leaves of maize, but this approach is only suitable for seedlings due to the seedling stage's few leaves and the sparser spacing between them. The majority of earlier research was conducted indoors, where the environment is steady, and picture acquisition and post-processing are simpler. There has not been any relevant information on LFW estimation. Numerous studies, including those on cotton [27, 28], rice [29], wheat [30], corn [31], and peanuts [32], have estimated the leaf area index. The outcomes assist breeders in making effective variety selections and offer growers precise field management options that boost crop yields.

In the estimation of soybean leaf parameters, A high-resolution RGB, multispectral, and thermal imaging multisource data technique based on unmanned aerial system (UAS) acquisition was developed by Maimaitijiang M. et al. [33] to estimate the soybean LAI. Throughout the growing season, they gathered RGB, multispectral, and thermal imaging photos of crops. From these images, they extracted vegetation indexes and crop surface models (CSMs) to create vegetation cover extraction models. Then, image parameters and models were combined to predict the soybean LAI using the partial least squares regression (PLSR), support vector regression (SVR), and extreme learning machine for regression (ELR) techniques. However, the optimal function of various crop varieties or leaf parameters varies from study to study, even within the same study [34–37], showing that simple regression models are insufficient for model generalizability when estimating crop leaf parameters. Most studies that use regression models for leaf parameter estimation achieve satisfactory accuracy.

With the ongoing development of sensor and image processing technologies, machine learning—an essential area of computer science—is now extensively applied in all facets of precision agriculture research, including leaf dynamic monitoring [38]. Moreover, machine learning methods are more precise and effective than conventional linear regression models and have been frequently utilized to create prediction models to link image data and biological parameters [39]. There have been few soybean studies to explore the prediction effect of various machine learning models on the leaf parameters of a single plant throughout its entire growth period. This is because the model prediction effect varies for different crops and environmental parameters.

Simple nonlinear regression (SNR) is a nonlinear regression function that has unknown regression coefficients as input. Generally, nonlinear regression occurs when the regression law is graphically represented as various curves with different shapes, and the dependent variable of the regression model is a function of the independent variable more than once. Common nonlinear regressions include hyperbola models, quadric models, logarithmic models, trigonometric models, exponential models, power function models, reduced order generalized integrator (ROGI) curves, modified exponential growth curves, etc. In many practical problems, regression functions tend to be complex nonlinear functions, so they are widely used.

Breiman first presented the random forest (RF) in 2001. Its ensemble machine learning approach is built on numerous categorical regression trees [40]. The fundamental idea of RF is to bootstrap aggregation and grow a decision tree in each subset of the training dataset to produce a homogeneous subset (number of trees: ntree). By averaging all decision trees, the RF's final outcome is obtained [40]. Repetitive sampling-related overfitting can be successfully reduced via RF regression [41].

In 2017, the Russian search engine giant Yandex released the categorical boosting (Cat Boost) algorithm, a machine learning library that is a member of the boosting algorithm family. Cat Boost is a novel machine learning algorithm framework based on gradient boosting decision trees (GBDT). In contrast to conventional neural network models, Cat Boost can adapt to training and high-precision diagnosis under small-scale data and does not need a large number of samples as a training set. Its benefits include overcoming gradient bias, effectively resolving the issue of prediction bias, increasing the algorithm accuracy, enhancing the model generalizability, and preventing overfitting [42–44].

The development of non-destructive estimation technology has enabled efficient and accurate monitoring of soybean leaves, which can meet the needs of breeders and is expected to be added to the routine monitoring indicators of breeding to serve efficient breeding. To improve the efficiency of acquiring biological traits for soybeans and speed up the breeding process, this study aims to evaluate the accuracy and generalizability of machine learning regression models in the prediction of soybean leaf parameters and select the best model for the dynamic estimation of soybean growth using the phenotypic features extracted from RGB images.

## Results

### High correlation image parameter selection

**Fig. 1:**
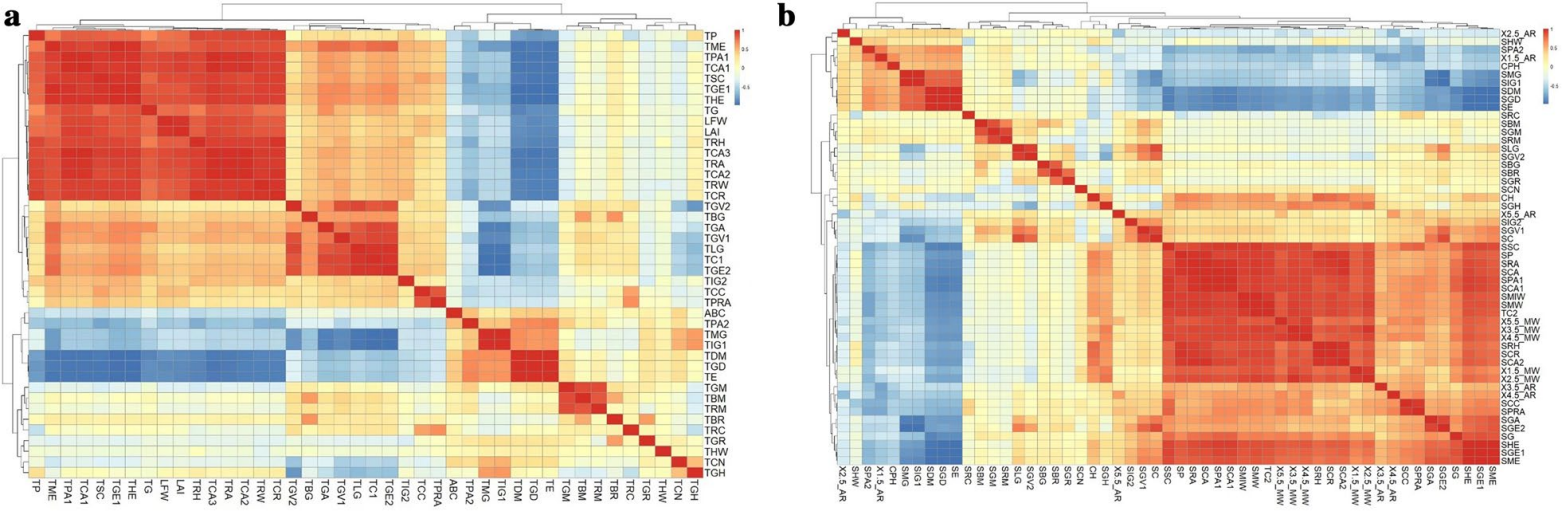
Heatmap of soybean leaf parameters correlated with image parameters

The heatmap analysis shows the correlation of leaf parameters with the top (Fig. 1a) and side (Fig. 1b) image parameters. Red indicates a positive correlation and blue indicates a negative correlation. The greater the correlation is, the darker the color. The correlation analysis of leaf parameters and 39 top image parameters showed a high correlation between the parameters. Among them, there was a positive correlation between LN, LFW, LAI, and 26 indexes, such as TBG and TBR (Specific definitions are provided in Additional file 1 and Additional file 2), and a negative correlation with 13 indexes, such as TBM and TGM. The correlation analysis results of leaf parameters and 53 side image parameters showed that a positive correlation between LN and 36 indexes, such as SBM and SBG, and a negative correlation with 17 indexes, such as SGM and SRM. Unlike that of LN, LFW and LAI are negatively correlated with SBM and SBR.

In addition, the correlation between the top and side image parameters has high similarity; for example, there was a high correlation between SCA1 and LN, and there was also a high correlation between TCA1 and LN. SPA1 had a high correlation with LFW, and TPA1 had a high correlation with LFW. Therefore, it is interesting to further explore the relationship between the side and top image parameters and soybean leaves.

Of the 92 image markers, SCA1, SPA1, SSC, TCA1, TPA1, and TSC had the strongest correlation to LN, LFW, and LAI. Six image parameters were involved in this case, three of which were side image parameters (SCA1, SPA1, and SSC) and three of which were top image parameters (TCA1, TPA1, and TSC). As in the previous example, the side image parameters matched the top image parameters one-to-one. From Table 1, it is clear that there is a strong correlation between the three side image parameters and LN, LFW, and LAI. The correlation coefficients between SCA1 and SPA1 and the leaf parameters, in particular, ranged from 0.872 to 0.952, from 0.872 to 0.951, and those between SSC and the leaf parameters ranged from 0.888 to 0.931.

**Table 1:** Correlation between soybean leaf Parameters and image parameters (Top6)

Index	LN	LFW	LAI
SCA1	0.872^**^	0.939^**^	0.952^**^
SPA1	0.872^**^	0.939^**^	0.951^**^
SSC	0.888^**^	0.925^**^	0.931^**^
TCA1	0.803^**^	0.883^**^	0.894^**^
TPA1	0.804^**^	0.882^**^	0.893^**^
TSC	0.806^**^	0.883^**^	0.894^**^

^**^indicates P < 0.01

### Simple nonlinear regression performance

Three characteristics, SCA1, SPA1, and SSC, were used as the main parameters for simple nonlinear regression since there is a strong association between the soybean leaf parameters and these characteristics. The polynomial quadratic function and exponential function models were investigated in depth using tenfold cross-validation. The findings indicated that the polynomial quadratic function model had an average R^2^ value between 0.77 and 0.92 and an ATPA value between 43.21% and 60.15%. The average R2 value in the exponential function model was between 0.77 and 0.91, and the ATPA value was between 51.34% and 73.99% (Table 2). Larger R^2^ and ATPA values were obtained for the LAI-SCA1 and LAI-SPA1 models (referred to as y-x). When compared to those of the dependent variables, the LAI estimates were the most accurate, with mean ATPAs, LN, and LFW values of 64.88%, 50.39%, and 51.23%, respectively. The best prediction accuracies for LN, LFW, and LAI were 55.14%, 55.95%, and 73.99%, respectively. The SCA1 image index had the highest prediction accuracy for the three leaf characteristics among the independent variables. The polynomial quadratic function and exponential function models were not significantly different in terms of R^2^. However, better ATPA values were obtained using the exponential function, with a distribution range of 50.94–73.99%.

**Table 2:** Results of simple nonlinear regression with tenfold cross-validation

Dependent varfable	Independent variable	Model type	Model paramenters			MAE	R^2^	ATPA (%)
			a	b	c			
LN	SCA1	A	7.23 ± 0.38	0.10 ± 0.01	−5.05 ± 0.73	30.12	0.77	49.92
	SCA1	B	0.36 ± 0.01	0.82 ± 0.21	–	30.05	0.77	55.14
	SPA1	A	6.92 ± 0.39	0.09 ± 0.01	−4.95 ± 0.76	30.01	0.77	51.02
	SPA1	B	0.35 ± 0.01	0.82 ± 0.12	–	30.04	0.77	55.01
	SSC	A	−11.38 ± 0.28	4.45 ± 0.08	0.03 ± 0.01	28.63	0.79	47.46
	SSC	B	1.83 ± 0.12	1.29 ± 0.41	–	28.45	0.79	50.94
LFW	SCA1	A	−2.43 ± 0.30	0.05 ± 0.02	1.79 ± 0.31	9.17	0.88	43.21
	SCA1	B	0.03 ± 0.00	1.07 ± 0.22	–	9.93	0.88	55.95
	SPA1	A	−2.56 ± 0.30	0.05 ± 0.01	2.27 ± 0.39	10.23	0.88	43.57
	SPA1	B	0.03 ± 0.01	1.07 ± 0.03	–	9.93	0.88	55.57
	SSC	A	−4.50 ± 0.30	1.11 ± 0.05	0.04 ± 0.01	9.38	0.89	48.99
	SSC	B	0.23 ± 0.04	1.67 ± 0.12	–	6.25	0.89	51.34
LAI	SCA1	A	−405.78 ± 1.40	10.46 ± 0.01	0.01 ± 0.00	2242.61	0.91	59.90
	SCA1	B	4.57 ± 0.32	1.12 ± 0.04	–	2158.91	0.91	73.99
	SPA1	A	−433.34 ± 1.60	10.38 ± 0.01	0.01 ± 0.00	2257.25	0.91	60.15
	SPA1	B	4.38 ± 0.26	1.12 ± 0.15	–	2161.84	0.91	73.95
	SSC	A	−556.15 ± 17.93	184.22 ± 2.23	13.10 ± 0.01	2063.22	0.91	57.03
	SSC	B	43.68 ± 5.43	1.75 ± 0.13	–	2031.49	0.92	64.31

RMSE, R^2^, and ATPA represent the average prediction accuracy; Model A and Model B represent the polynomial quadratic function (y = a + bx + cx^2^) and exponential function (y = ax^b^), respectively

**Fig. 2:**
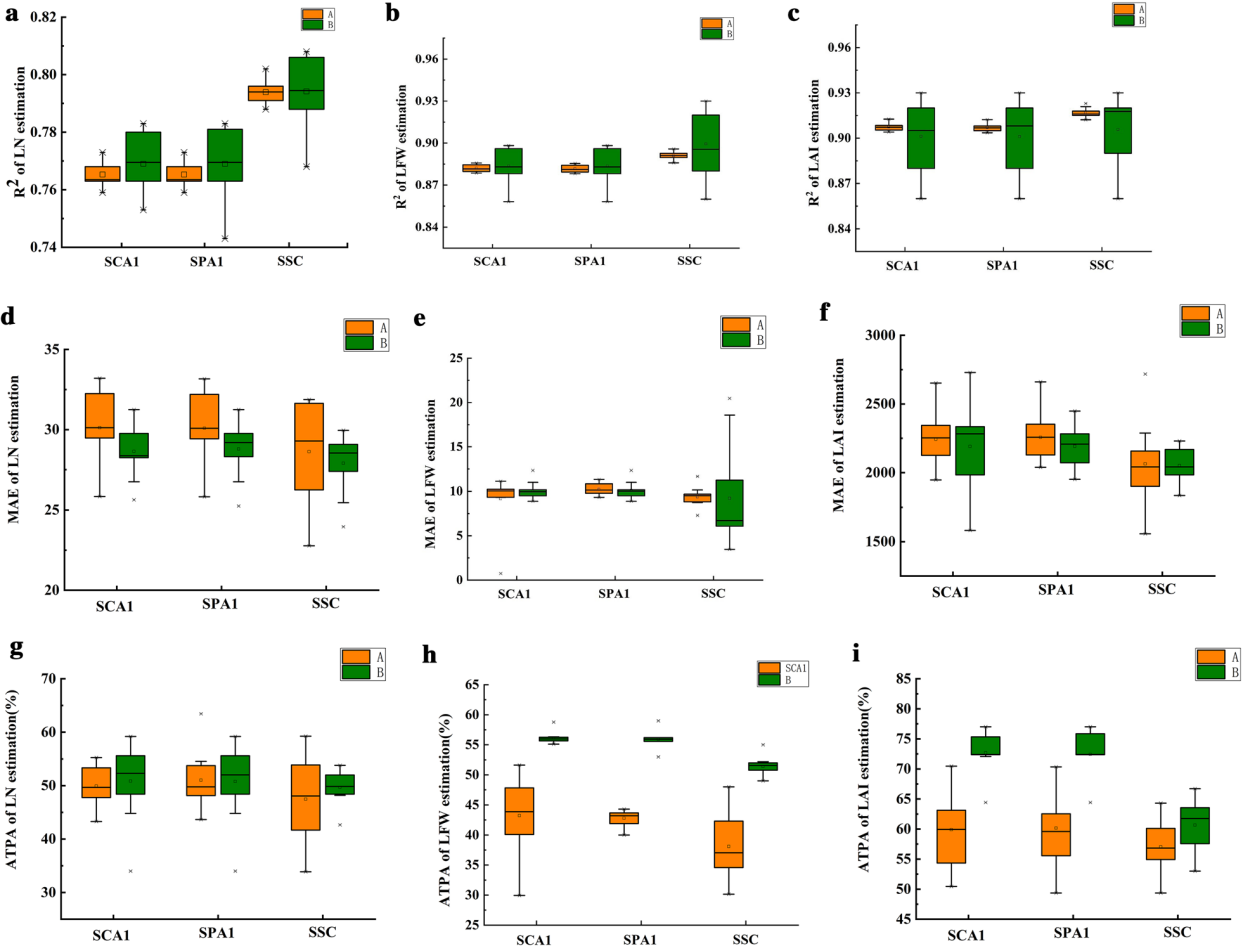
Prediction results of simple nonlinear regression on soybean leaf parameters. A represents the polynomial quadratic function model (y = a + bx + cx^2^), and B represents the exponential function model (y = ax^b^)

As seen in Fig. 2, compared with the results obtained using Model A (polynomial quadratic function), Model B had larger R^2^ and ATPA values and a lower MAE value. Both models had generally higher ATPAs for the estimate of soybean leaf parameters SCA1 and SPA1 than for those of SSC.

### Important parameter selection

When the four key predictors were retained, it was observed that the cross-validation curve demonstrated a reduction in error, leading to the best regression results. Following this, the predictors were ranked in order of their assigned significance value, and the top four predictors were selected as the optimal input parameters for the random forest model (Additional file 3: Figure S3). The use of these four indicators as input parameters resulted in a well-functioning forecast, as evidenced by the results presented in Table 3.The results showed that in the prediction of LN, the use of the first four predictors (SSC, SPA1, 2/5 MW(Additional file 3: Figure S4), and SCA1) was more effective than using all predictors. According to %IncMSE, the overall interpretation rate of the predictors (the 4 significant predictors) on the variance of the response variable LN in the model increased from 81.16% to 85.57%. This is due to the exclusion of unimportant or noisy predictors. Moreover, the actual number of leaves was more in line with the expected value, and the prediction performance was better. The overall interpretation rates of the variance associated with the response variables (LFW and LAI) by the predictors (the four major predictors) in the model reached 94.13% and 94.35% in the predictions of LFW and LAI, respectively, which was not significantly different from the previous values of 94.97% and 94.83%, respectively.

**Table 3:** Important parameter filtering

Predictors	Parameters number	Number of trees	Mtry	MSR	R^2^
LN	92	500	31	1659.642	0.8116
	4	300	21	1255.347	0.8557
LFW	92	500	31	167.0929	0.9497
	4	300	19	264.5167	0.9413
LAI	92	500	31	9250809	0.9483
	4	300	14	11554815	0.9435

### Random forest performance

**Fig. 3:**
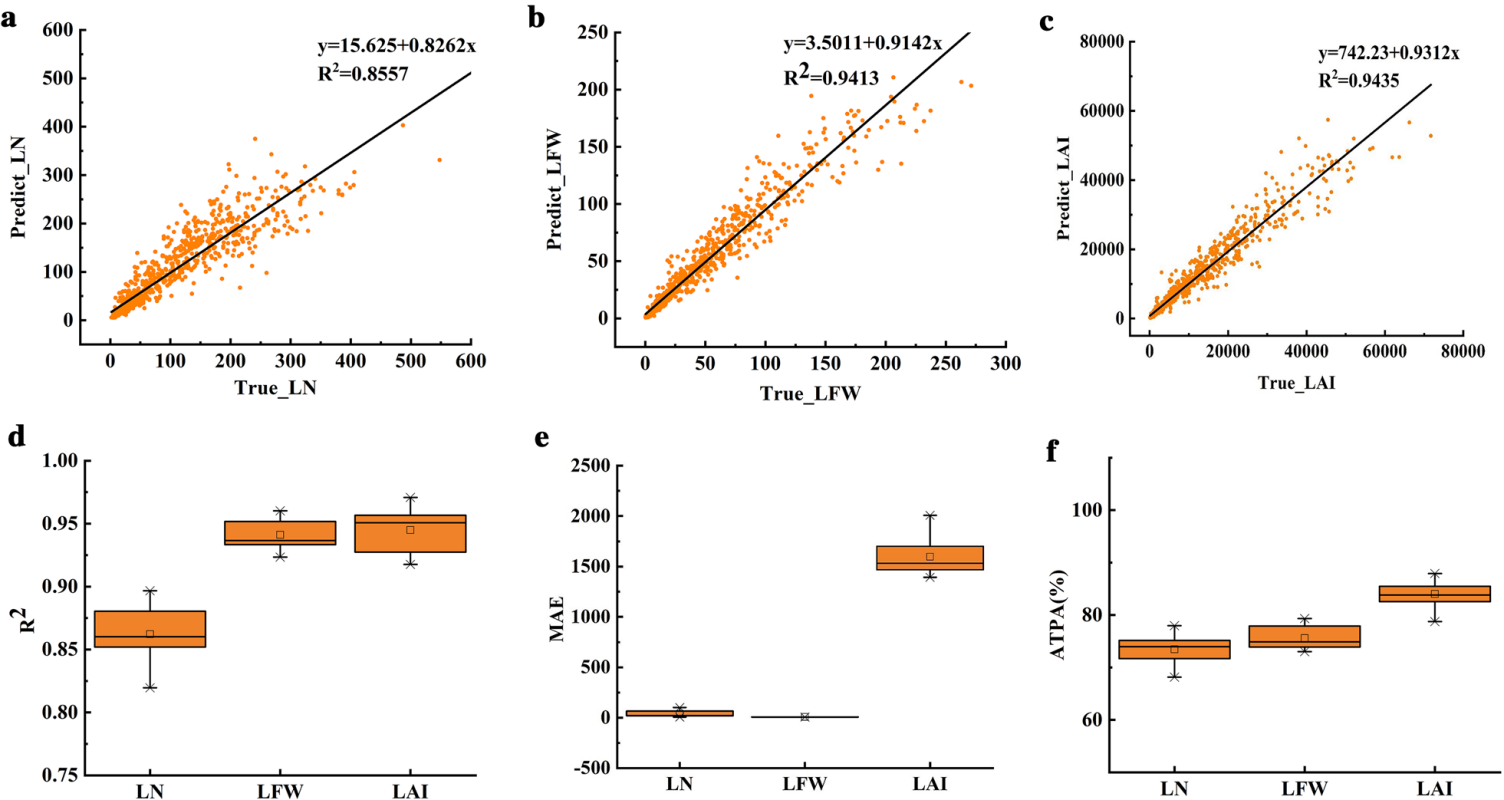
Soybean leaf parameter estimations based on the RF model. **a**–**c** show the fitting relationship between the predicted and true values of leaf parameters based on the RF model, and **d**–**e** show the R^2^, MAE, and ATPA values of the RF model under tenfold cross-validation

**Fig. 4:**
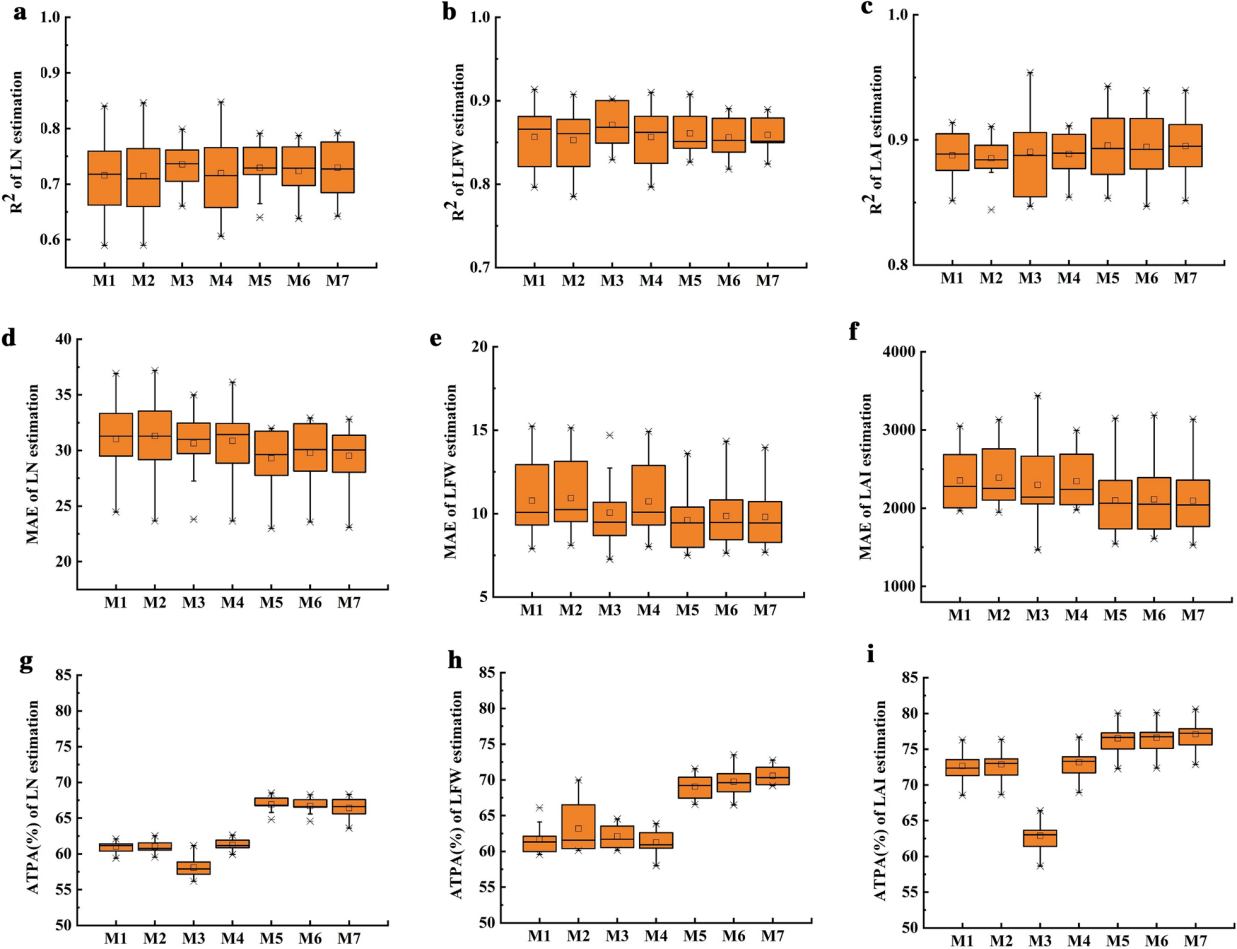
Soybean leaf parameter estimation based on the Cat Boost model. The R^2^, MAE, and ATPA values are the result of tenfold cross-validation. The x-axis labels represent different input variables for Cat Boost M1-M7. M1: SCA1; M2: SSC; M3: SPA1; M4: SCA1 + SPA1; M5: SCA1 + SSC; M6: SPA1 + SSC; and M7: SPA1 + SCA1 + SSC

The RF model was constructed using the four most crucial factors as the input variables, and tenfold cross-verification was carried out. Figure 4 depicts the R^2^, MAE, and ATPA distributions. In general, the RF model estimated all soybean leaf parameters with good accuracy. The predicted value was linearly fitted with the true value, and all points were closely and evenly distributed near the fitted line (Fig. 3a–c). The R^2^ values of LN, LFW, and LAI were 0.8557, 0.9413, and 0.9435, respectively, indicating that the prediction effect was good. According to the results of the tenfold cross-validation (Fig. 3d–f), the R^2^ distribution ranges of LN, LFW, and LAI were 0.81–0.89, 0.93–0.96, and 0.91–0.97, the MAE distribution ranges were 5.57–100.01, 5.06–7.40, and 1392.17–2006.45, and the ATPA distribution ranges were 68.14–77.97%, 72.77–79.33%, and 78.74–89.93%, respectively. Figure 4 shows that there were no outliers in terms of this model's R^2^, MAE, and ATPA predictors, demonstrating the model's good generalizability. At the same time, R^2^ and ATPA were consistent. They were both the lowest in terms of LN and the highest in terms of LAI. From Additional file 3: Figure S2, it can be seen that the importance of SSC is higher than that of other indicators, and LFW and LAI have high similarity, i.e., SSC > SCA1 > SPA1 > SPA2 for both.

### Input variable selection

Based on the SNR and RF results above, SCA1, SPA1, and SSC are highly correlated and important parameters. Therefore, they were selected as input parameters to combine as input variables. In this study, the Cat Boost model was used to estimate soybean leaf parameters, and 21 input combinations were trained and evaluated (three leaf parameters, LN, LFW, and LAI, as output variables, multiplied by seven different combinations of image parameters as input variables). The seven combinations of input variables are named M1-M7, where M1: SCA1; M2: SSC; M3: SPA1; M4: SCA1 + SPA1; M5: SCA1 + SSC; M6: SPA1 + SSC; and M7: SCA1 + SSC + SPA1.

### Cat boost performance

**Fig. 5:**
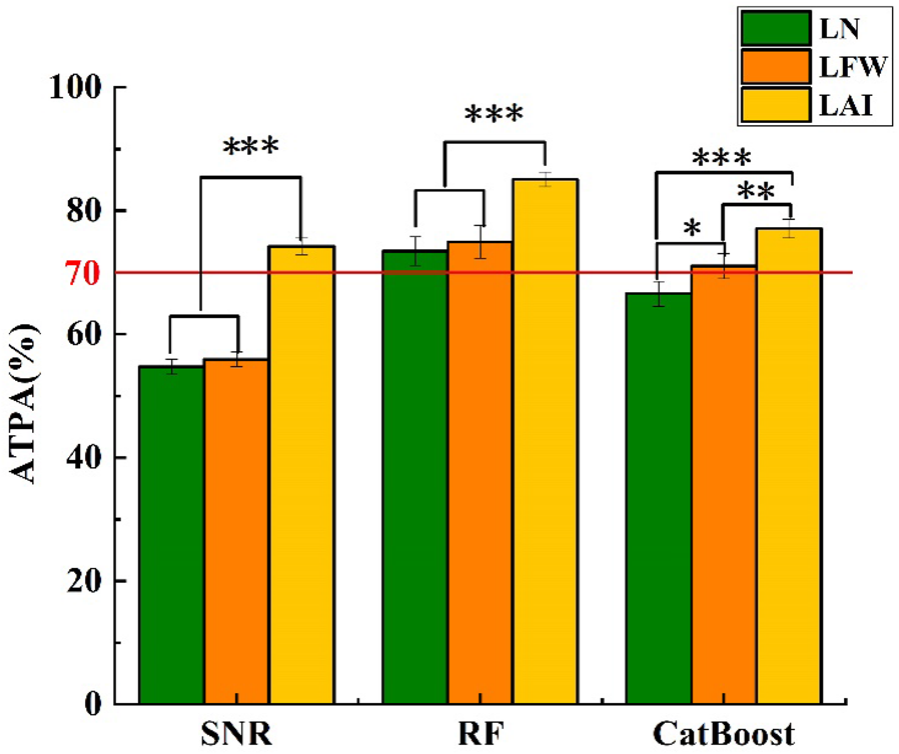
Comparison of the best prediction effect for the three models. The red line indicates ATPA = 70%

As shown in Fig. 5, in general, the Cat Boost regression model achieved high accuracy in estimating the three soybean leaf parameters. As the number of input variables increased, the MAE decreased, while the R^2^ and ATPA increased. For both LN and LAI, it was shown that M4, M5, M6, and M7 were more accurate than M1, M2, and M3 as input parameters. For LFW, M5, M6, and M7 were used as input parameters, and they had higher accuracies than those of M1, M2, and M3. For M1-M3, M2 was used as an input parameter and was more accurate than that of M1 and M3. As a result, the model prediction accuracy will rise when SSC is included in the input parameters. SSC is more significant than SPA1 and SCA1 (Additional file 3: Figure S5).

Moreover, Fig. 4 demonstrates that when M7 was utilized as the input parameter, there were no outliers in any of the prediction groups. When M7 was used as the input parameter for LN estimation, the R^2^, MAE, and ATPA distribution ranges were 0.67–0.78, 23.08–32.80, and 66.54–68.03%, respectively. When M7 was used as the input parameter for LFW estimation, the R^2^, MAE, and ATPA distribution ranges were 0.83–0.88, 7.68–13.95, and 69.94–73.81%, respectively. When M7 was used as the input parameter for the LAI estimation, the R^2^, MAE, and ATPA distribution ranges were 0.88–0.94, 1529–3137, and 71.43–80.51%, respectively. The largest average R^2^ values for LN, LFW, and LAI as well as the highest ATPA values were 0.73, 0.86, and 0.89, respectively, in the Cat Boost regression model estimate.

### Comparison of the best prediction effect for the three models

The three regression models were combined to determine which had the best predictive power for soybean leaf characteristics (Fig. 5). The three models achieved the highest tenfold cross-validation prediction accuracies for LAI, with scores of 74.21%, 85.09%, and 77.09%, respectively, and all > 70%. The overall ATPA performance was RF > Cat Boost > SNR. The RF ATPAs for LN, LFW, and LAI reached 73.45%, 74.96%, and 85.09%, respectively, which were 6.93%, 3.98%, and 8.01%, respectively, higher than those of the optimal Cat Boost model and 18.78%, 19.08%, and 10.88%, respectively, higher than those of the optimal SNR model.

### Prediction effect of soybean leaf parameters under RF model

As shown in Table 4, based on the RF model, the soybean leaf parameters under net cropping and sleeve cropping were predicted, and in the MC model, the distribution range of R^2^ was 0.88 ~ 0.96, the distribution range of MAE was 5.06 ~ 1841.17, and the distribution range of ATPA was 73.12% ~ 83.21%. In IC mode, the distribution range of R^2^ is 0.86 ~ 0.96, the distribution range of MAE is 7.01 ~ 1820.51, and the distribution range of ATPA is 70.98% ~ 84.10%. It can be seen that the RF model has high prediction accuracy in both planting modes.

**Table 4:** Prediction effect of soybean leaf parameters in two planting modes under RF model

Dependent varfable	Planting method	Independent variable		
		R^2^	MAE	ATPA (%)
LN	MC	0.88	54.22	73.12
	IC	0.86	58.79	70.98
LFW	MC	0.95	5.06	77.79
	IC	0.94	7.01	75.98
LAI	MC	0.96	1841.17	83.21
	IC	0.96	1820.51	84.10

MC: soybean monoculture; IC: maize‒soybean relay strip intercropping**.** R^2^, MAE and ATPA are averages

**Fig. 6:**
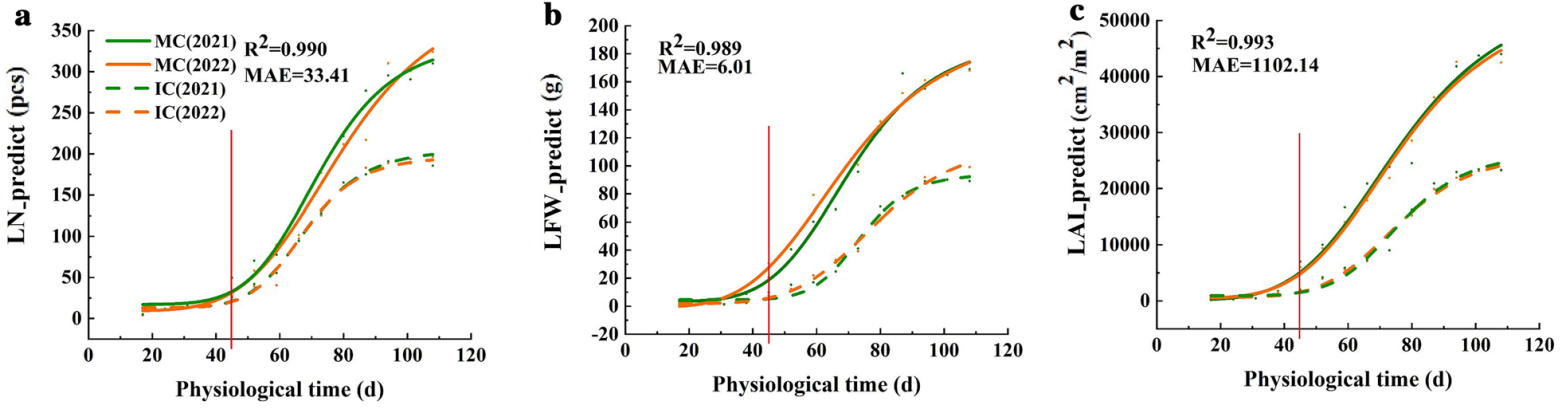
Growth dynamics of soybean leaf parameters under the RF model. Take one of the varieties, for example. MC: soybean monoculture; IC: maize‒soybean relay strip intercropping. The red line indicates the time of the last image collection before the maize harvest, and both R^2^ and MAE are averages

Given that the soybean leaves showed a steady upward trend throughout the whole growth period, the logistic function model was able to forecast the growth dynamics of these leaves accurately. The RF model was used to generate LN, LFW, and LAI growth curves, and the results are presented in Fig. 6. The R^2^ values for these curves were 0.990, 0.989, and 0.993, respectively, showing that the model could be used to accurately depict soybean growth dynamics. In general, shading inhibited increases in LN, LFW, and LAI that were difficult to overcome in later growth stages, and there were obvious differences between the two treatments. The increases in LN, LFW, and LAI all showed a gradual increasing trend as the reproductive period progressed. The maize was harvested 45 days after soybean sowing. The soybeans return to a normal light environment, where LN was most sensitive and increased rapidly, and LFW and LAI showed rapid increasing trends 1 week after reillumination. For the two treatments, LN, LFW, and LAI under nesting reached their peaks before the net cropping, but the rapid growth periods of LFW and LAI under the set cropping were approximately 1 week later than that of the next net cropping. It is clear that the RF model is able to compare the variations in soybean growth rules under various treatments, which is important for real-world applications.

## Discussion

### Soybean leaf parameter prediction based on RGB images

Because of their straightforward operation, portability, variety of applications, affordability, and wealth of image data, RGB cameras are frequently utilized in machine vision applications. The most accurate LAI forecasts using data fusion with RGB cameras and thermography was obtained by Mai Maiti Jiang et al. [45]. Based on RGB images captured by unmanned aerial vehicles (UAVs), Roth L et al. [46] estimated soybean LAI with an R^2^ of 0.89 and an RMSE of 0.41 m^2^ m^−2^. According to Bai G et al. [22], the automatic scoring of IDC was accomplished using RGB images gathered under field conditions, image processing, and machine learning technology. The overall classification accuracy was  > 81%. As a result, the spectral and structural data from RGB images, along with machine learning technologies, were used to track soybean leaf growth parameters during the whole growth phase.

### Performance of the three models

Machine learning is a subfield of artificial intelligence that is extremely popular due to its superior capacity to combine complicated and dynamic biological knowledge with enormous amounts of omics data. Several predictive models and decision-making algorithms can be constructed using machine learning approaches [47]. The R^2^, MAE, ATPA, and other metrics are used to assess a model’s estimated influence; in general, the higher the R^2^ and ATPA values are, the smaller the MAE value, and the better the results. It does, however, compared to R^2^ and MAE, ATPA has a strong ability to distinguish models with similar performance levels [48]. The main purpose of this research is to compare and contrast three distinct regression techniques using ATPA. The RF regression model is the most accurate and stable of the three compared models.

In previous studies, the majority of the input parameters are provided directly, and the source and justification for parameter selection are not disclosed [49], which makes readers wonder about the precision and importance of the selected parameters. To provide input parameters with a foundation and increase reading comprehension and the model prediction precision, this work not only introduces an extraction procedure for 92 image parameters but also screens and combines them.

The dependent variable for the SNR model is chosen based on the image parameters with the highest association among the 92 image parameters (39 top image parameters and 53 side image parameters) and the soybean leaf parameters (LN, LFW, and LAI). The findings demonstrate a high correlation between the soybean leaf parameters and the top and side image parameters, which is likely a result of the correlation between the top and side of the same indicator. For example, SSC reflects the compactness rate of soybean plants on the side, while TSC reflects the compactness of soybeans at the top, representing different viewing angles. In addition, among the dependent variables, LAI is the most accurately estimated, with a mean ATPA of 64.88%. Among the independent variables, comparing the polynomial quadratic and the exponential function models, it can be seen that the R^2^ and ATPA values of Model B (exponential function) are generally higher than those of Model A (polynomial quadratic function), and the MAE value is generally lower than that of Model A. For both models, the higher the correlation with soybean leaf parameters is, the higher the ATPA. For example, the correlation between SCA1, SPA1, and LAI is as high as 0.952 and 0.951, and the ATPA is 73.99% and 73.95% when predicted by the exponential function. This is probably because the computational relationship between the independent factors and the variables in simple nonlinear regression models is quite straightforward and is not difficult to train.

The RF model selects input parameters based on the significance of each image parameter. The findings reveal that, with distribution ranges of 68.14–77.97%, 66.77–79.33%, and 78.74–89.93%, respectively, better predictions of LN, LFW, and LAI were made in terms of ATPA. These benefits of RF models are primarily attributable to the incorporation of multiple ML algorithms (such as bootstrap aggregation and random variable selection), which reduce overfitting and autocorrelation of the input variables [42] and generally have no negative impact on the model when more input variables are added [50]. As a result, the RF model has strong generalizability [48] and the highest prediction accuracy. Input parameter filtering and model hyperparameter adjustment have greatly increased the prediction accuracy in this work [45] in comparison to that in earlier research findings. The LAI, which is the same as the SNR, prediction accuracy is best for the RF model. The RF model in Fig. 5, on the other hand, shows that SSC, as opposed to the SNR, is more significant than the other indicators.

The results demonstrate that excellent accuracies in terms of the three soybean leaf parameters are achieved. The Cat Boost regression model uses the three image parameters with the strongest correlation and relevance as input parameters. The best average R^2^ values for LN, LFW, and LAI as well as the highest average ATPA values were 0.73, 0.86, and 0.89, respectively, for the Cat Boost regression model. As the number of input variables increases in each combination, R^2^ and ATPA increase, but MAE decreases. We also find that when SSC is present in the input parameters, the prediction accuracy of the model increases, which means that SSC plays an important role in the model prediction, which is the same result as that found for RF. Overall, the average ATPA of the three regression models is RF > Cat Boost > SNR.

Does the prediction accuracy increase with the number of input parameters? No. Due to the high collinearity between a large number of input parameters, the R^2^ value in the RF prediction model improves or does not change much when the results using the 4 key parameters are compared to those using the 92 image indicators as input parameters. We need more training data and a preliminary study of the input variables to mitigate the impact of autocorrelation in this case [49]. With more input parameters, the Cat Boost model prediction accuracy increases. It is clear that the filtered indicators have a positive influence on the model prediction.

### Future direction

The results of this study show that adding more input parameters does not always result in improved predictions; in fact, it can occasionally decrease the prediction accuracy. As a result, before making predictions, the input parameters must be analyzed. Improved prediction performance can be achieved by performing a correlation analysis between the projected target qualities and image metrics or by choosing input metrics that are of high importance. Additionally, there are three categories of image parameters: morphology, color, and texture. Morphological parameters can only be extracted from a binary value map, whereas color parameters must be drawn from a finely segmented color map, and texture parameters must be drawn from a grayscale map. Therefore, the extraction of various kinds of parameters will result in a heavy workload and high hardware facility requirements. If we can determine the optimal input parameters for each target trait through continuous practical verification, we can greatly reduce the upfront workload and improve the work efficiency. The five input parameters (SCA1, SPA1, SSC, SPA2, and 2/5 MW) used in the three regression models in this study are morphological parameters in the image parameters. This is a very satisfying finding because it means that more focus can be placed on morphological parameters in the subsequent study of soybean leaf parameters, which will significantly lessen the workload of future researchers.

This article presents 11 soybean varieties, 2 treatments, and 2 years of experimental data, which is not sufficient to support the creation of a strong model. Thus, by utilizing information from additional soybean variety and years, the ideal model, input parameters, and model hyperparameters can be found for forecasting soybean leaf parameters.

## Conclusion

The segmentation of RGB images is accurate, and the IOU, PA, and recall are as high as 0.98, 0.99, and 0.98, respectively, based on the RGB camera used in this study to track the soybean leaf index throughout the entire growth cycle.

Three regression methods (SNR, RF, and Cat Boost) were evaluated to estimate three soybean leaf parameters (LN, LFW, and LAI) in different growth environments (soybean monoculture and maize‒soybean relay strip intercropping). The three parameters with the highest correlation were used as input parameters in the SNR model; the four parameters with the largest importance were used as input parameters in the RF model; and the three parameters with the strongest correlation and importance were combined as input parameters in the Cat Boost model. All of these parameters are morphological parameters. The results demonstrated that RF > Cat Boost > SNR in terms of ATPA for the three regression models. The RF ATPAs for LN, LFW, and LAI reached 73.45%, 74.96%, and 85.09%, respectively, which were 6.93%, 3.98%, and 8.01%, respectively, higher than those of the optimal Cat Boost model and 18.78%, 19.08%, and 10.88%, respectively, higher than those of the optimal SNR model. Hence, RF was the best model for soybean leaf parameter estimation based on the phenotypic traits extracted from RGB images. Thus, it is possible to precisely depict the growth curve, which has the potential to speed up the soybean breeding process.

## Materials and methods

### Test site overview and experimental design

This study was conducted in 2021–2022 at the Chongzhou Experimental Base of Sichuan Agricultural University (103° 39′ E, 30° 33′ N). The area has a humid subtropical monsoon climate with an average annual temperature of 16.2 °C, 1400 h of sunlight, and 918 mm of rainfall. The basic chemical properties of the 0–20 cm soil layer at the test site were as follows: soil organic matter content: 24.3 g/kg, total potassium 15.2 g/kg, total nitrogen 1.6 g/kg, total phosphorus 1.3 g/kg, available potassium 169.4 mg/kg, available nitrogen 299.5 mg/kg, and available phosphorus 36.5 mg/kg.

**Fig. 7:**
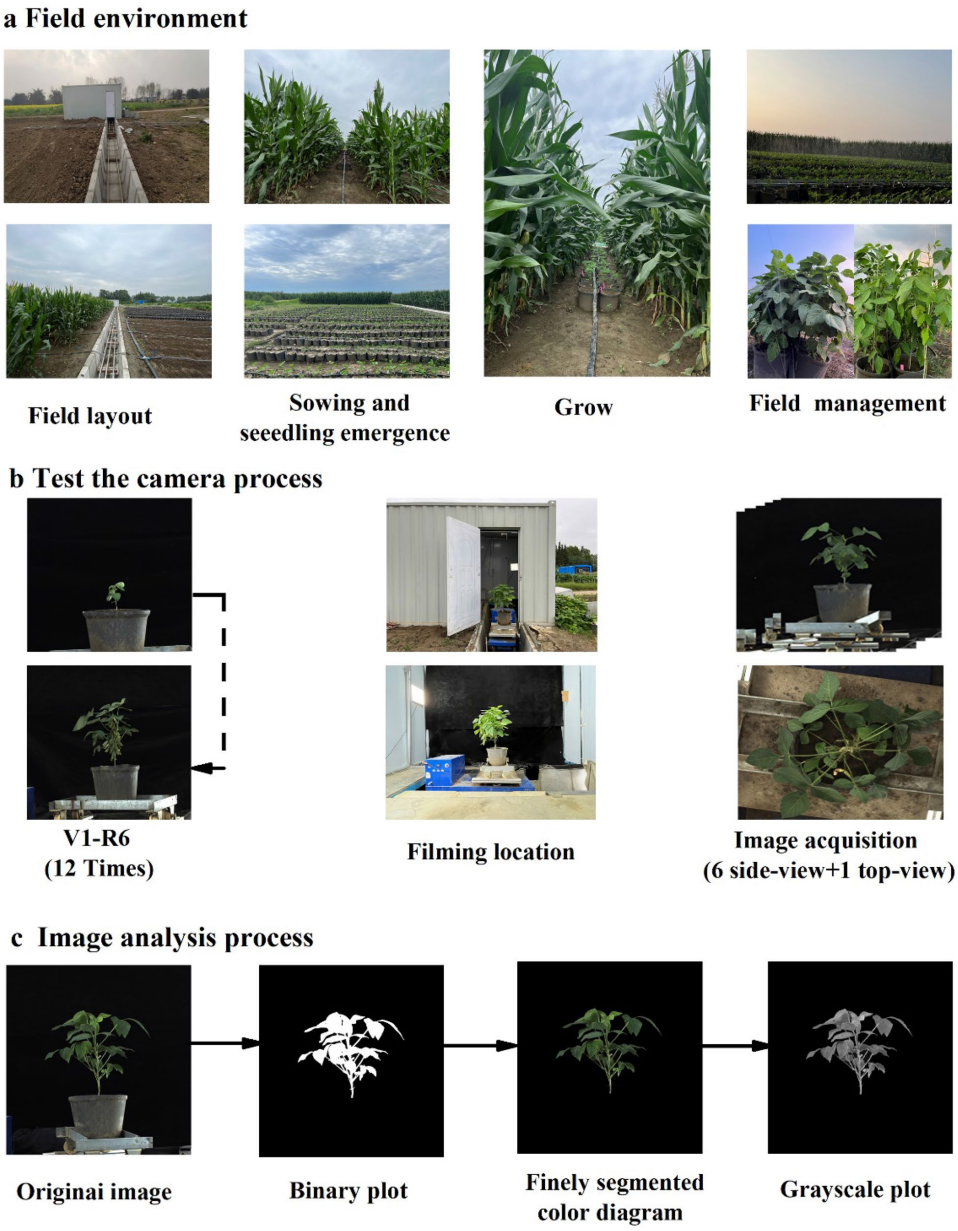
Soybean high-throughput phenotype

Eleven soybean cultivars (Five varieties were planted in 2021 and six more varieties were added in 2022), each with three replicates and two planting methods (soybean monoculture and maize‒soybean relay strip intercropping), were used in the test. Small-scale spring maize variety Zhongyu 3 was used for the maize–soybean interplanting strategy. The Engineering Technology Research Center of Crop Strip Compound Planting, Department of Agronomy, Sichuan Agricultural University, provided the planting supplies. Figure 7a displays the field configuration, which has a belt length of 20 m and a bandwidth of 2 m. For maize‒soybean relay strip intercropping, two rows of maize (the maize belt) were placed within two rows of soybean (the soybean belt). The row spacing of maize‒maize and soybean‒soybean was 40 cm, and the spacing between the maize belt and soybean belt was 60 cm. Both maize and soybeans were sown in single plants with a hole spacing of 20 cm. The soybeans were planted in pots with a top diameter of 25 cm, a bottom diameter of 20 cm, a height of 25 cm, and 10 kg of soil in a long row of maize under relay strip intercropping, with two pots planted side by side in each belt. When growing soybeans in a monoculture, the plants and their row spacing matched those in relay strip intercropping. The bottom fertilizer of maize was 923 kg·hm^−2^ of compound fertilizer (N:P:K = 13:5:7), and 98 kg hm^−2^ and 163 kg hm^−2^ of urea (N ≥ 46%) were applied at the jointing and corn pumping stages, respectively. The soybeans were not fertilized during the whole growth period.

### High-throughput phenotype acquisition

During the whole growth period of soybeans in 2021–2022, we used the single plant soybean imaging platform independently developed by Sichuan Agricultural University. The main body of this platform is an automatic rotary table, with industrial cameras installed on the top and side. The rotary table sets the rotation speed and number of rotations with a programmable logic controller (PLC) controller. Hikvision industrial cameras (MV-CH250-90GC, Hangzhou, China), each with a Hikvision robot lens (MVL-KF1624M-25MP, focal length 16 mm, maximum aperture F2.4, 1.2’’ C-mount lens, Hangzhou, China), were used as the sensors. The following camera settings were used for collecting images. The top and side camera focal lengths were 2.3 mm and 2.4 mm, respectively, Images were collected at distances of 2.6 m and 1.8 m, respectively, Camera mode set to Aperture Priority (AV), aperture size of 2.4; this resulted in a camera frame rate of 4.5 fps. JPG files of sizes 3680*4360 and 5108*4604 were used to store the side and top photos, respectively. We obtained ruler images of the top and side views using a white standard plate with a diameter of 30 cm, and we utilized these ruler images to determine the value of the extracted picture characteristics.

The sampling dates are shown in Table 5. The first image collection date was from the soybean V1 period. For image acquisition, 3 pots in each process were selected randomly. The soybean plants were placed on the rotary table. Side images were collected every 60° rotation, and the top view was randomly captured. Finally, 1 top view and 6 side views were captured for each soybean plant. A total of 2160 side view photos and 360 top view photos were taken. The specific shooting process is shown in Fig. 7b.

**Table 5:** Sampling dates for the experiments

Years	Transplanting date	Sammpling dates	Number of samples
	(month-day)	(month-day)	
2021	6–19	7–3(V1)、7–10、7–26、8–3、8–10、8–17、	672
		8–24、8–31、9–7、9–14、9–24、9–31	
2022	6–9	6–26(V1)、7–3、7–10、7–17、7–24、7–31、	1848
		8–7、8–14、8–21、8–28、9–4、9–11	

V1 indicates the period when soybeans grow their first three leaf complex

The soybean plant segmentation model uses UNet. The 2520 images collected on 24 sampling dates in 2021 and 2022 were preprocessed, including image screening (2480 representative images were selected from the 2520 images taken as samples), image resizing (the image was resized to 2048*2048), and manual annotation (using Photo Shop to annotate images with pixel-level accuracy to obtain black and white labeled images). The 2480 images after preprocessing and labeling are divided into training set, test set and validation set according to the ratio of 8:1:1. The VOC2007 dataset was used to pre-train the network and obtain the weights. Transfer learning using pre-trained weights, formal training is divided into two phases. The first stage freezes the weights of the backbone feature extraction network and strengthens the feature extraction network, and only trains the classification network. The second stage is to unfreeze the feature extraction network and train the entire network. and the specific training parameters are shown in Table 6.

**Table 6:** UNet model training parameters

Stage	Learning rate	Epoch	Batchsize	Learning rate decay
1	1*10^–4^	30	2	0.9
2	1*10^–5^	50	1	0.95

*represents a multiplication sign

The specific processing steps for feature extraction (Fig. 7C) are as follows:

1. The preprocessed image is input into the trained UNet to obtain a binary map. After obtaining the binary plot, the results need to be evaluated. In semantic segmentation tasks, common evaluation methods are the intersection over union (IOU), pixel accuracy (PA), and recall (Recall). Among them, IOU reflects the degree of coincidence between the predicted result and the real result, which is the most important indicator, PA reflects the probability that each pixel in the image is classified correctly, and Recall reflects the proportion of the target area that is correctly recognized. The labels in the test set are compared with the prediction results of the Unet network to calculate the IOU, PA, and Recall of each graph. Average all images in the test set. The calculation formulas are as follows:
  1 $$IOU=\frac{\mathrm{TP}}{\mathrm{TP}+\mathrm{FP}+\mathrm{TN}}$$
  2 $$PA=\frac{\mathrm{TP}+\mathrm{TN}}{\mathrm{TP}+\mathrm{FP}+\mathrm{TN}}$$
  3 $$\mathrm{Recall}=\frac{\mathrm{TP}}{\mathrm{TP}+\mathrm{TN}}$$

TP represents a pixel that is actually a plant pixel and is judged by the network to be a plant; FP represents a pixel that is actually a plant pixel but is judged by the network to be background; TN represents a pixel that is actually a background pixel and is judged by the network to be background; and FP represents a pixel that is actually a background pixel but is judged by the network to be a plant.

The IOU, PA and Recall of the VGG16-Unet model used in this paper are as high as 0.98, 0.99, and 0.98, which are very close to the manually annotated images and can meet the needs of program analysis.

(B) To create a precisely segmented color map, the preprocessed picture and binary map mask are combined. The following is the calculation formula:
  4 $$imag{e}_{roi\left(x,y\right)}=\left\{\begin{array}{c}(\mathrm{0,0},0), imag{e}_{binary\left(x,y\right)}=0\\ imag{e}_{orginal\left(x,y\right)}, imag{e}_{binary\left(x,y\right)}=1\end{array}\right.$$

where (x,y) is the coordinate of a certain pixel, image_roi refers to the segmented color image, image_binary is the binary image output by UNet, and image_orginal is the original color image. image(x,y) is the value of the pixel in the image with coordinates (x,y).

(C) The color space of the accurately segmented color map is converted to the HIS color space, and the I channel is used as the grayscale map. The calculation formula is as follows:
  5 $$I=\frac{R+G+B}{3}$$
(D) The OpenCV library is used to process the image. The NumPy library is called for data calculation, and the independent programming code is used to extract the feature values of the soybean image.Forty-six morphological features were retrieved based on binary images; 12 color features were retrieved based on finely segmented color images; and 34 texture characteristics were retrieved based on grayscale photos. The image feature values were determined by averaging the feature values from the six side views and top view. In Additional file 1, the parameters are categorized, and in Additional file 2, the characteristic parameters are described.

### Traditional phenotype acquisition

After image acquisition, the plants were destructively sampled to obtain manual agronomic parameters. Specifically, the plants with collected image information were destructively sampled, the soybean leaves were picked off and photographed, the number of leaves was counted with Image-Pro, the leaf area was extracted, and finally, the weighing record was recorded. LAI is the ratio of leaf area to floor space.

### Soybean leaf parameter prediction

To better estimate soybean leaf parameters, including LN, LFW, and LAI, three different regression methods, SNR, RF, and Cat Boost, were used. Tenfold cross-validation was performed on the data collected from 24 sampling dates in 2021 and 2022, and R^2^, the mean absolute error (MAE), and ATPA were used to evaluate the performance of each estimation method. ATPA is calculated as follows:

6 $$\mathrm{ATPA}=\left(1-\frac{1}{N}{\sum }_{i=1}^{N}\frac{\left|{T}_{A}-{T}_{P}\right|}{TA}\right)*100$$

### Simple nonlinear regression (SNR)

Prior to performing a regression analysis, the image parameters and soybean leaf parameters (LN, LFW, and LAI) were compared using the Pearson correlation analysis. The image parameters with the highest correlation coefficients were then chosen for further investigation. A straightforward nonlinear regression model was selected because, as Additional file 3: Figure S1 demonstrates, there is a nonlinear relationship between the two variables. The significance of the p value in the output indicates that the second-order polynomial is the most significant when using the poly function to identify the order of the best multinomial regression equation. As a result, the polynomial quadratic function (Eq. 7) and exponential function (Eq. 8) were carefully chosen.

7 $${\text{y}}\, = \,{\text{a}}\, + \,{\text{bx}}\, + \,{\text{cx}}^{2}$$

8 $${\text{y}}\, = \,{\text{ax}}^{{\text{b}}}$$

### Random forest (RF)

The training set is used for the preliminary training of the random forest model, whereas the test set is used to assess the effectiveness of the model training. Not all 92 image features make a significant contribution to the regression accuracy, and certain image index features are not readily apparent, which may cause much noise and lead to high model accuracy errors. Thus, low-contribution image metrics must be eliminated. The importance function in the Sklearn library was used in this study to assess each variable's significance using "%IncMSE" as the assessment index. The average total nodal impurity reduction (also known as the average impurity reduction or Gini coefficient importance) for all the trees in the entire forest was used to calculate the importance of the input variable [41, 51, 52]. The importance of each variable was calculated as a percentage of the total contribution of all the variables in the model, where the total importance of all the variables was 100. Each predictor was rounded against the result from the tenfold cross-validation curve. This curve suggests that retaining n significant predictors is the best possible regression result because the error is reduced. Hence, the predictors were ordered from high to low according to the determined value of each predictor's relevance, and then the top n predictors were chosen as input parameters into the random forest model.

To determine the optimal number of trees (ntree) estimated by soybean LN, LFW, and LAI, the tree values were tested in increments of 50 from 100 to 500, and a value of 300 trees was chosen because stable and low MAE and higher R^2^ and ATPA were achieved for all 3 leaf parameter estimation models. Other hyperparameters in RF regression were set to default values according to the regressor function in the scikit-learn library.

### Cat boost

As input variables for the Cat Boost model, the SNR- and RF-based study findings were combined with a variety of measures that have the highest correlation and relevance. To reduce prediction error and boost prediction accuracy, modification parameters were also applied. The learning rate was set to 0.04, the loss function was set as the RMSE, the number of iterations was set to 2000, and the other hyperparameters were set to their default values.

### Data analysis

RGB images were segmented using UNet, and image parameters were extracted with Python 3.7 (Python Software Foundation, https://www.python.org/) and the scikit-learn module version 0.21.3. Using the R-based statistical modeling package named anomaly, 68 sets outliers were removed from 2520 sets of data using the test function in the vehicle package, and the remaining 2452 sets of data were used for the analysis. The model generalizability was examined using tenfold cross-validation, and the R^2^, MAE, and ATPA distributions (interval, median, and mean ranges) were employed for the model evaluation to lessen the impact of data segmentation on the model estimation error. To create the figures, RStudio and Origin 2018 was used.

## Supplementary Information

**Additional file 1.** Thumbnail Table of Image Parameters.

**Additional file 2.** Definitions and Calculation Formulas of Image Parameters.

**Additional file 3.** Supplementary Illustration Figure.

## Data Availability

The datasets used in this study is available from the corresponding author on reasonable request.
