# Supplementary material for: Soybean leaf estimation based on RGB images and machine learning methods
Source: Plant Methods. 2023 Jun 17;19:59. doi: 10.1186/s13007-023-01023-z (PMC10276400; doi:10.1186/s13007-023-01023-z)
Supplement: Supplementary file 3 — Additional file 3. Supplementary Illustration Figure. [file 13007_2023_1023_MOESM3_ESM.docx]

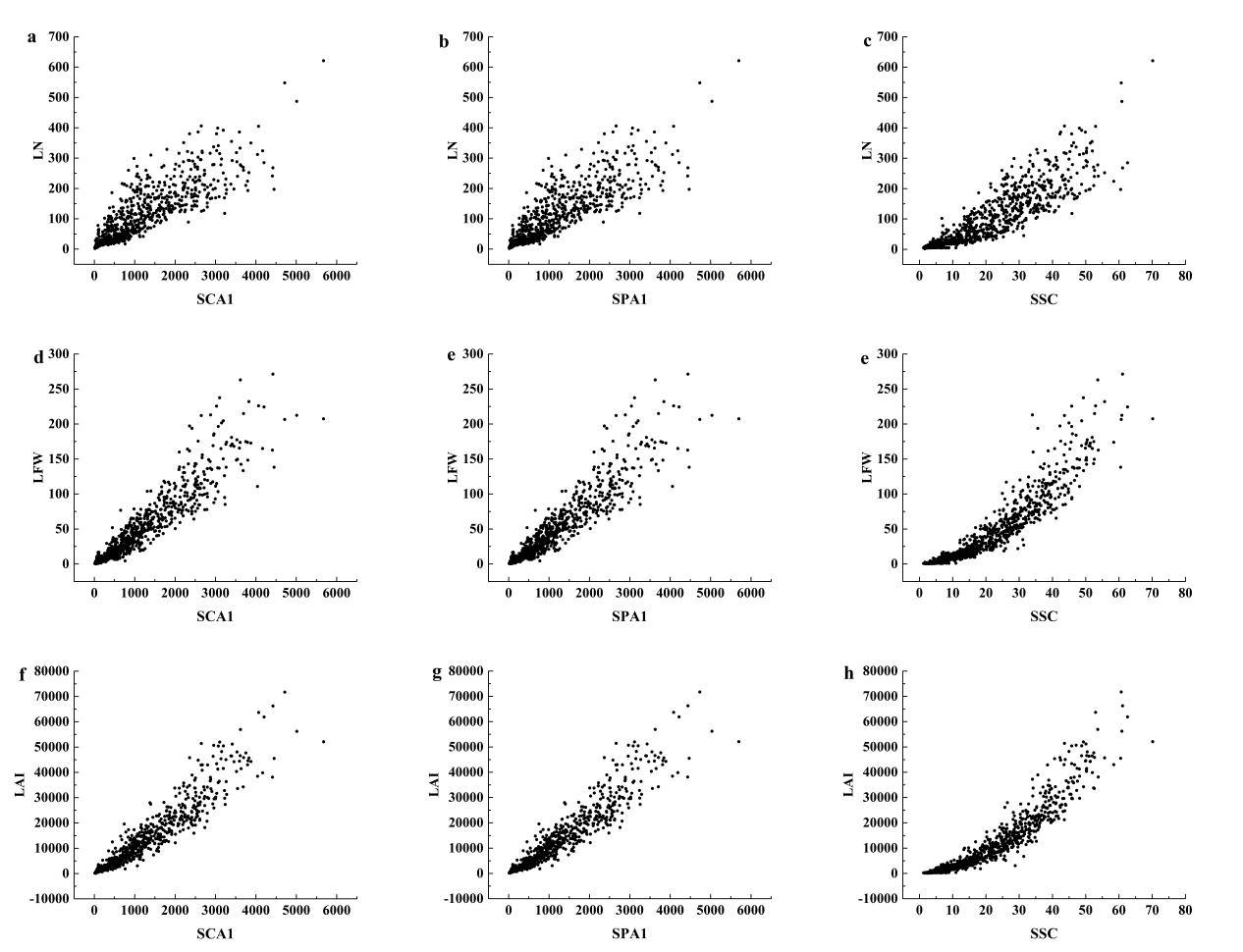


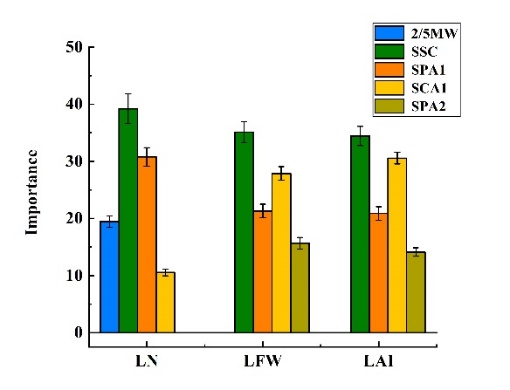
S1. Relationship between highly correlated image parameters and soybean leaf parameters

S2. The importance of each input parameter in the RF model


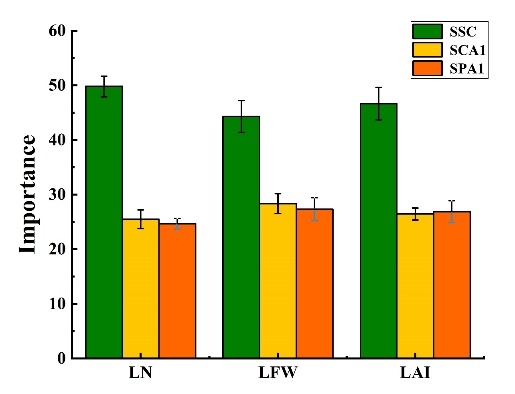


S3. The importance of each parameter in the M7 combination in the Cat Boost model


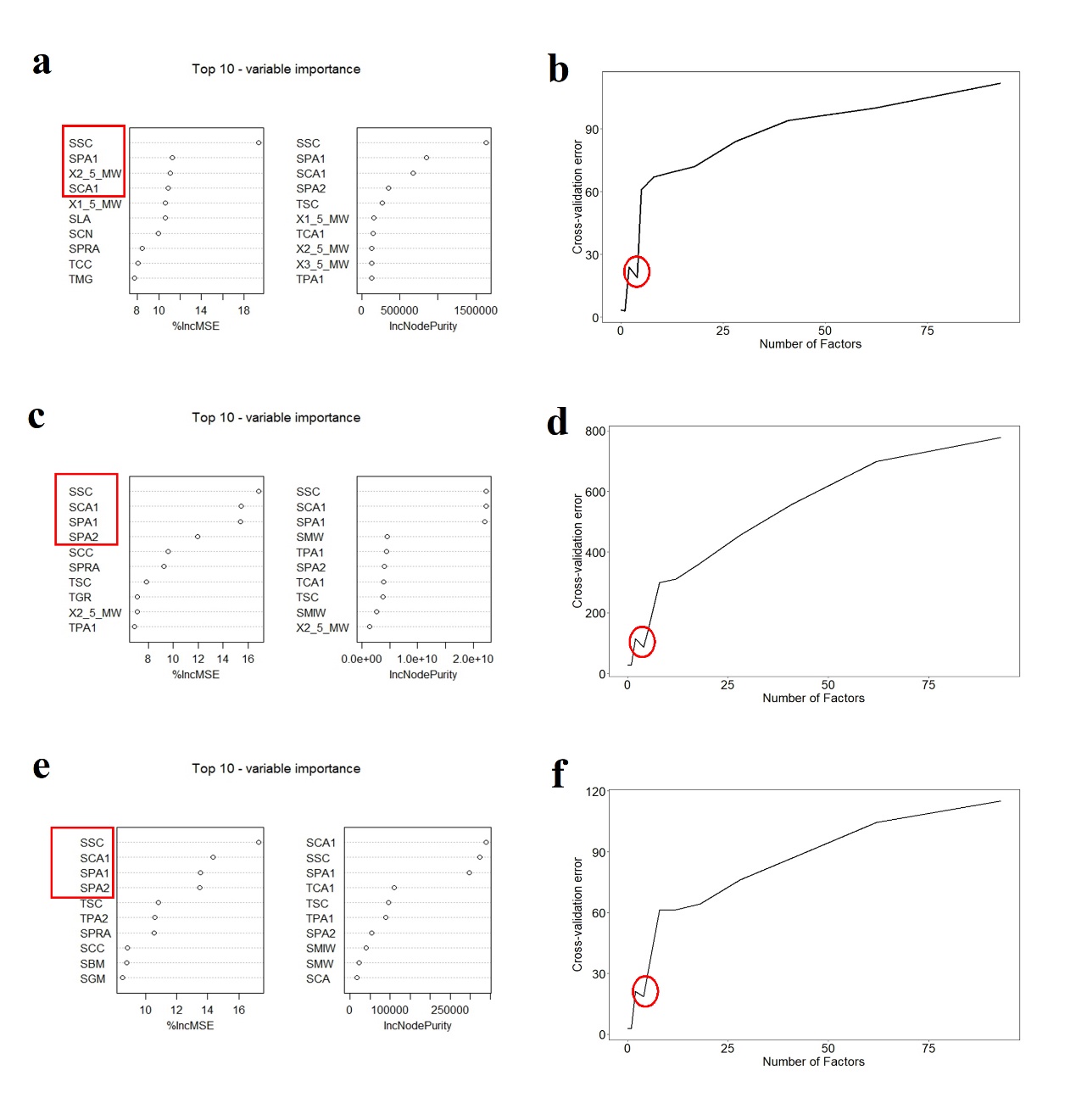
S4. Screening of important indicators under ten-fold cross-validation. Fig a shows the importance of each index when predicting LN (Top10), and Fig b shows the relationship between the number of input indicators and the error when predicting LN. Fig c shows the importance of each index when predicting LFW (Top10), and Fig d shows the relationship between the number of input indicators and the error when predicting LFW. Fig e shows the importance of each indicator when predicting LAI (Top10), and Fig f shows the relationship between the number of input indicators and the error when predicting LAI. The red rectangle represents the selected parameter, and the red circle marks the number of parameters with a small error.


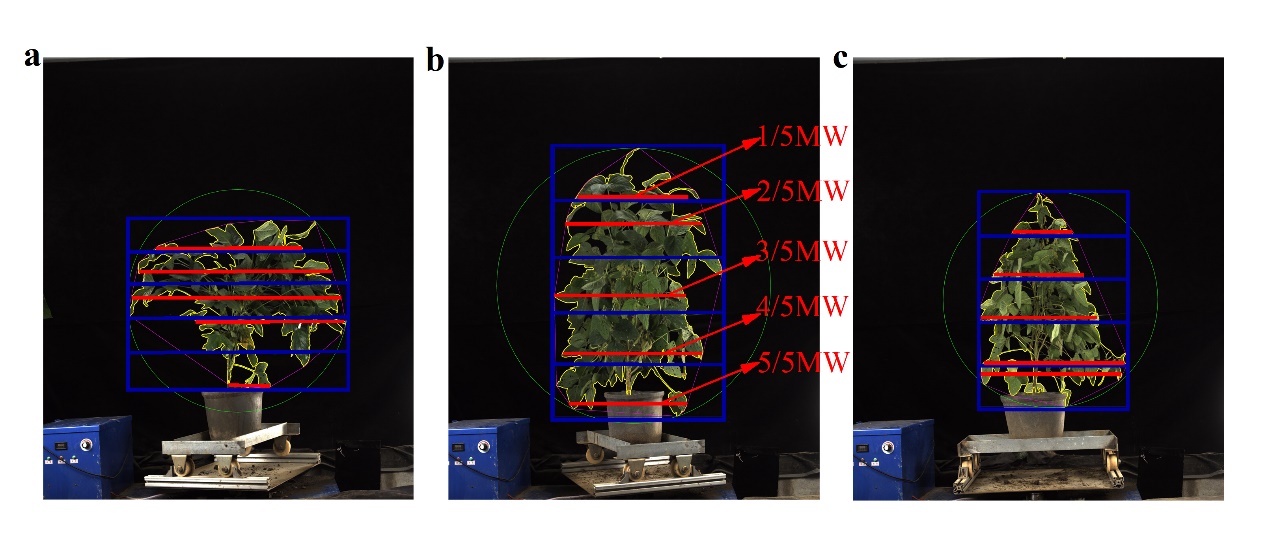
S5. Image parameter annotation (partial). The blue border in the figure indicates that the soybean plant is divided into 5 parts from top to bottom. The red lines represent the maximum width of each section.

Coefficients:

Estimate Std. Error t value Pr(>|t|)

(Intercept) 21.20 10.67 1.988 0.047115 *

poly(SCC, 3, raw = TRUE)1 -299.77 113.04 -2.652 0.008130 **

poly(SCC, 3, raw = TRUE)2 1355.27 355.26 3.815 0.000145 ***

poly(SCC, 3, raw = TRUE)3 -977.28 340.61 -2.869 0.004203 **

---

Signif. codes: 0 ‘***’ 0.001 ‘**’ 0.01 ‘*’ 0.05 ‘.’ 0.1 ‘ ’ 1

Residual standard error: 35.67 on 985 degrees of freedom

Multiple R-squared: 0.4654, Adjusted R-squared: 0.4637

F-statistic: 285.8 on 3 and 985 DF, p-value: < 2.2e-16
